# Feasibility of abdominal fat quantification on MRI and impact on effectiveness of abdominal compression for radiotherapy motion management

**DOI:** 10.1016/j.tipsro.2023.100232

**Published:** 2023-12-19

**Authors:** M. Daly, L. McDaid, C. Nelder, R. Chuter, A. Choudhury, A. McWilliam, G. Radhakrishna, C.L. Eccles

**Affiliations:** a Division of Clinical Cancer Sciences, School of Medical Sciences, Faculty of Biology, Medicine and Health, University of Manchester, Northern Ireland, United Kingdom; b Department of Radiotherapy, The Christie NHSFT, Wilmslow Road, Manchester M20 4BX, Northern Ireland, United Kingdom; c Department of Medical Physics and Engineering, The Christie NHSFT, Wilmslow Road, Manchester M20 4BX, Northern Ireland, United Kingdom; d Department of Clinical Oncology, The Christie NHSFT, Wilmslow Road, Manchester M20 4BX, Northern Ireland, United Kingdom

## Abstract

The impact of fat on abdominal compression effectiveness in abdominal cancers was determined using magnetic resonance imaging (MRI). Visceral and subcutaneous fat were delineated on T2W 3D MRI, and motion change with compression was measured on 2D cine MRI. Results from 16 participants showed no correlation between fat percentage, body mass index (BMI), and motion change. Median BMI was 28.7 (SD, 4.9). Mean motion reduction was 7.8 mm (IQR, 5.0; p = 0.001) with compression. While no direct link was found between fat, BMI, and compression effectiveness, abdominal compression remains crucial for motion management in radiotherapy planning, providing dosimetric benefits.

## Highlights

• MRI fat quantification is novel in the context of radiotherapy motion management.
• Abdominal compression significantly reduced respiratory motion in the majority of patients.
• BMI and abdominal adipose tissue were not found to impact compression effectiveness.

## Introduction

Radiation doses to the upper abdomen are limited by nearby critical organs at risk (OAR) like the duodenum [1]. A significant challenge of abdominal stereotactic ablative body radiotherapy (SABR) is respiratory motion, impacting planned dosimetry [2], [3], [4], [5]. One radiotherapy motion management strategy is abdominal compression [2], [5]. However, compression may not be suitable for all patients as no reduction or even increased motion with compression has been seen, particularly smaller magnitudes (<5 mm) [6] and, it may not be tolerated due to discomfort [7].

It is unknown which patients benefit most from abdominal compression, so prospective assessment of factors affecting effectiveness warrants evaluation. High body mass index (BMI) has been suggested as a factor limiting effectiveness, with abdominal adipose tissue (AT) acting as a cushion attenuating the compression force [8], however, there is no data published supporting this. BMI is used to measure adiposity but does not account for muscle mass and fat distribution [9], and has limitations across ages, sexes, and ethnicities [9], [10]. Single-slice fat quantification on MRI may be an alternative predictor for radiotherapy motion management strategy as it could be performed prospectively on pre-treatment diagnostic imaging [11].

BMI and abdominal AT have not been evaluated in the context of abdominal compression effectiveness for respiratory motion management. The aims of this proof-of-principle study were to 1. assess the feasibility of single-slice percentage AT volume (%AT) quantification on MRI acquired using standard clinical sequences acquired using an integrated magnetic resonance linear accelerator (MR Linac), and 2. identify any associations between subcutaneous adipose tissue (SAT) and visceral adipose tissue (VAT) and abdominal compression effectiveness.

## Methods & materials

Adult participants enrolled with informed consent in two ethics-approved non-interventional observational studies were eligible for inclusion in this evaluation: QUANTUM (NCT04748094 [12]), a single-centre study, and PRIMER (NCT02973828 [13]), a multi-centre national study. Non-patient staff healthy volunteers (HV), and patient volunteers undergoing radical upper abdominal radiotherapy were included. HV were identified through institutional email, and patients through the relevant multidisciplinary meeting.

Participants were imaged on a 1.5 T Unity MR Linac (Elekta, Crawley, UK), images were acquired in free breathing and then repeated directly after with an abdominal compression belt (Freedom^TM^ Belt, CDR Medical Systems, Calgary, Canada). Compression was applied to the upper abdomen inferior to the xiphisternum, according to local protocol, ensuring a taut fit, and inflated to the maximum level of individual tolerance. As part of this protocol, no minimum/maximum pressure levels are set. Participants fasted for ≥2 h prior to imaging, were positioned head-first supine on the MR Linac couch and scanned with either arms up or by sides based on comfort.

T2-weighted (W) turbo spin-echo (TSE) images were acquired with the following parameters: echo time (TE) 70 ms, repetition time (TR) 1187 ms, flip angle = 90°, field of view (FOV) 450 × 399 mm, in-plane resolution 2.0 × 2.0 mm, slice thickness 3 mm. Image FOV was selected to include the diaphragm, liver, and radiotherapy treatment site. Total image duration was 2 m 46 s. T2W images were selected as they are routinely acquired for pancreas patients in our institution due to increased inherent tissue contrast of OARs.

A single-slice balanced steady-state free procession gradient echo (bFFE) cine-MRI was used for motion quantification. For the initial 7 participants (2 HV, 5 patients), single-slice coronal cines were acquired for sequence development with the following parameters: TE = 1.35 ms, TR = 2.7 ms, flip angle = 40°, FOV = 448 × 400 mm, matrix 132 × 150, pixel size 3 × 3 × 10 mm. Temporal resolution was 0.42 s, and 250 dynamics were acquired with a total acquisition time (TA) of 1 m 46 s. For the latter 9 participants bFFE cine-MRI sequences were acquired in all three orthogonal planes. Only the coronal plane was used for motion assessment in this study to ensure parity between all participants. Cine slice position was selected manually to cover: the central abdominal structures, and the approximate disease site for patients, or the head of pancreas for HV. These image parameters were: TE = 1.34 ms, TR = 2.7 ms, flip angle = 40°, FOV = 10 × 448 × 400 mm, matrix 132 × 150, pixel size 3 × 3 × 10 mm. Temporal resolution was 1.3 s, and 750 dynamics across all three planes were acquired with a TA of 5 m 18 s.

**Fig. 1:**
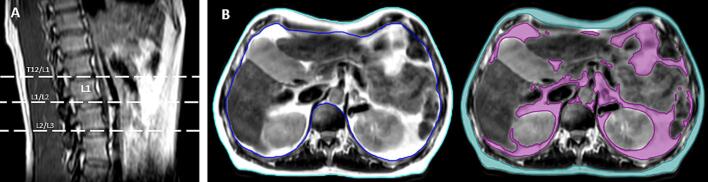
Example of delineation borders for A. anatomical points for measurement, and B. external patient contour (cyan), internal patient contour (blue), SAT (teal), and VAT (purple).

AT was delineated by a single observer (MD), using the Raystation (V12, Raysearch Labs, Stockholm, Sweden) system at three anatomical points: the intervertebral disc between the 12th thoracic vertebra (T12) and 1st lumbar vertebra (L1); between L1 and 2nd lumbar vertebrae (L2); and between L2 and the third lumbar vertebra (L3) (Fig. 1a). SAT and VAT were delineated on the free-breathing T2W scans, to avoid distortion of anatomy by the abdominal compression belt. SAT was manually delineated using the Smart Contour tool, and VAT using participant-specific automatic signal intensity thresholding, visual inspection, and manual editing where required.

External and internal contours were delineated: external defined as the external border of the patient surface, and internal defined as the internal border of the ribcage (Fig. 1b). Structure volumes were calculated automatically by the system and recorded. AT was defined as the percentage of internal/external contour attributable to fat: %AT = AT volume (cm^3^) divided by external/internal patient volume (cm^3^).

Motion was assessed using software (MATPEL, V3.9, Amsterdam, Netherlands) by rigid registration of the initial cine image to subsequent images, based on a 2-dimensional (2D) clip-box volume incorporating upper abdominal organs. The maximum range of peak-to-peak motion in the craniocaudal plane was recorded, and percentage change with abdominal compression was calculated.

Participant characteristics were summarised using descriptive statistics. No threshold for clinically relevant motion changes was set as any reduction in respiratory motion magnitude is assumed to be of benefit. For statistical analysis, the cohort was split into two groups by BMI according to the median value (BMI = 27.2) to assess the differences for change in motion magnitude with compression using a Mann-Whitney *U* test. Changes in motion magnitude with and without compression was analysed using a Mann-Whitney *U* test. Spearman rank-order correlation between percentage change with abdominal compression and variables including age, BMI, and SAT and VAT %AT at various points was evaluated. Analyses were conducted using Prism (V9.5.0, GraphPad Software Inc, Boston, USA) and Excel (V2211, Microsoft Corporation, Redmond, USA).

## Results

Between November 2019 to January 2021 (PRIMER, arms B & D) and May 2021 to October 2022 (QUANTUM, cohort 1), 16 participants were included in this study, their characteristics are outlined in Table 1. Three were HV, and 13 were patient volunteers with abdominal treatment sites including pancreas, liver, and adrenal gland. Median age of included participants was 61 years (range, 24–82), and most [12] were males. Mean BMI was 28.3 (SD, 4.8), with males being higher (29.43, SD 4.04) than females (26.74, SD 7.65).

**Table 1:** Participant characteristics, N = number.

			*N*	*%, range, SD*
**Participants *(N, %)***			16	100
	Healthy volunteers		3	18.8
	Patient volunteers		13	81.3

**Sex *(N, %)***				
	Male		12	75
	Female		4	25

**Age *(median, range)***				
	Years		61	24–82

**BMI *(mean, SD)***			28.3	4.8
	18.5–24.9		4	25
	25–29.9		7	43.8
	>30		5	31.3

**Treatment site *(N, %)***				
	N/A		3	18.8
	Liver		7	43.8
	Pancreas		4	25
	Adrenal		1	6.3
	Nodal metastasis		1	6.3

Most participants were either overweight or obese according to BMI (*n* = 12). %AT was calculated for all participants at all 3 measurement points (Table 2A), apart from participant 14, whose images did not extend inferior enough to delineate AT at L2/L3. Mean %AT differed between sexes (Table 2B), e.g., at L1/L2 mean (SD) SAT %AT values for females were higher at 27.52 % (14.1) than 20.81 % (6.9) for males, and mean (SD) VAT %AT at the same level were higher for males (50.68%, SD 12.79) than females (41.07, SD 17.08) respectively. However, due to the variation in numbers of both subgroups, this was not statistically tested. There was a moderate correlation between VAT at all three levels (T12/L1, L1/L2, L2/L3) and BMI (*r* = 0.63, 0.54, 0.59, respectively), but none between SAT and BMI at the same levels (*r* = 0.32, 0.06, −0.02).

**Table 2A:** A: Body mass index (BMI), percentage subcutaneous adipose tissue (SAT) and visceral adipose tissue (VAT) fat (%AT) results for all participants. Note: the imaging FOV for participant 14 did not extend low enough for AT quantification at the level L2/L3.

			SAT %AT			VAT %AT		
Participant		BMI	T12/L1	L1/L2	L2/L3	T12/L21	L1/L2	L2/L3
Healthy volunteers	1	28.4	16.5	21.6	27.3	41.9	53.1	63.2
	2	37.9	42.0	47.0	52.7	51.7	53.0	51.0
	3	30.1	29.6	34.9	40.9	61.8	64.4	64.9

Patient volunteers	4	27.4	13.2	13.7	15.7	37.8	32.8	33.9
	5	26.1	25.6	32.3	39.8	43.7	52.5	44.7
	6	32.9	14.3	14.9	19.9	78.2	74.6	80.6
	7	28.3	12.2	14.4	16.4	44.0	61.3	56.2
	8	25.3	19.7	24.7	28.8	42.2	53.7	42.8
	9	26.3	19.6	21.6	26.7	49.0	44.8	30.3
	10	26.9	13.9	15.9	18.8	31.4	44.2	56.8
	11	21.7	21.6	25.3	33.2	24.0	40.4	42.0
	12	24.3	10.9	14.8	22.2	24.7	32.4	37.7
	13	35.0	19.8	20.6	23.5	45.6	52.1	63.3
	14	35.1	21.0	21.4	–	48.0	57.3	–
	15	22.0	10.9	13.1	18.6	16.2	17.1	22.5
	16	24.4	18.7	23.5	28.0	46.6	38.7	34.7

	**Mean**	28.3	19.4	22.5	27.5	42.9	48.3	48.3
	**SD**	4.8	8.0	9.2	10.4	14.9	14.0	15.8
	**IQR**	5.7	7.4	10.0	11.6	12.1	14.7	23.8

**Table 2B:** Percentage subcutaneous adipose tissue (SAT) and visceral adipose tissue (VAT) fat volume results split by sex.

		T12/L1	L1/L2	L2/L3
SAT	Male	17.95	20.81	25.39
	Female	23.58	27.52	33.31

VAT	Male	46.05	50.68	51.48
	Female	33.54	41.07	39.57

**Fig. 2:**
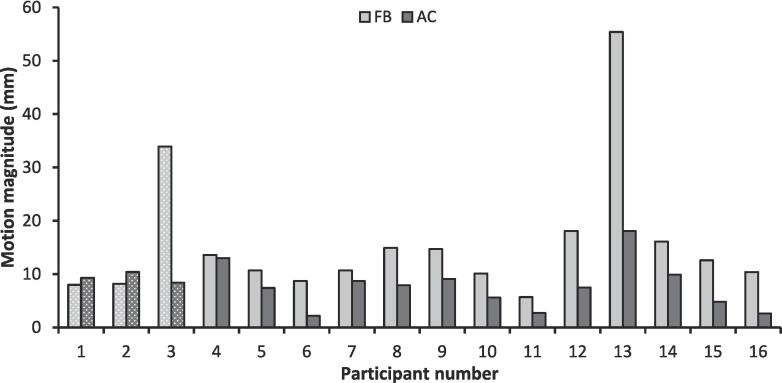
Bar chart showing the peak-to-peak motion results for each participant, free breathing (FB) and with abdominal compression (AC). Participants 1–3, shown with dotted shading, are healthy volunteers.

Motion results are shown in Fig. 2. Mean and interquartile range (IQR) of motion in free breathing were 15.7 mm (5.5) and 8.0 mm (4.1) with abdominal compression. Males had a larger mean initial motion of 17.5 mm (IQR, 6.3) than females (10.4 mm, IQR, 5.6). Overall, the mean change in motion was a 7.8 mm (IQR, 5.0 mm) reduction with compression, and the mean percentage motion change was a 40.3 % (IQR, 35.6%) reduction. Males showed a larger mean percentage motion reduction with compression (42.5%, IQR.52) than females (33.8 %, IQR 27.3). Motion increased with abdominal compression in two participants (both HV), with initial motion in free breathing of ∼8 mm. For HV, the mean percentage change in motion was 10.7 % (IQR, 51.3 %), and for patient volunteers 47.2 % (IQR, 23.6 %). Nine participants (1 HV, 7 patients) saw a reduction of ≥40 %. The change in motion with abdominal compression was significant for all participants (*p* = 0.001). There was no statistically significant difference in % motion change between the two BMI groups (*p* = 0.44), although mean % change was larger in the BMI < median group (51.2%) in comparison to BMI > median (29.4%). No correlation between percentage motion change and either SAT or VAT %AT were seen. BMI had only a weak correlation with percentage motion reduction (*r* = −0.31).

## Discussion

This is the first study to evaluate the association between abdominal fat on MRI and abdominal compression effectiveness for radiotherapy. This study showed single-slice %AT quantification to be feasible on routine MR images acquired for radiotherapy on an MR Linac. No association between change in respiratory motion with abdominal compression and SAT/VAT levels, or BMI, was found. However, in this study motion reduction was larger in patients with BMI less than 27.2, which may be clinically significant.

One of the limitations of this study was the small sample size, limiting statistical power. As ethnicity and sex impact both respiratory kinematics and fat distribution [14], [15], future studies should endeavour to conduct subgroup analyses. Intra-observer variation of AT delineation was not quantified for this work, and motion was quantified only in one plane. Finally, patient experience, tolerability and reproducibility of compression were not measured in this proof-of-principle feasibility study.

High BMI was suggested to affect compression effectiveness in one study in Shanghai, China [8], however no association was seen in a similar study in New York, USA [16]. Neither studies reported the ethnicity of included patients. Ethnicity affects local AT distribution [10], for example those of Chinese ethnicity demonstrate higher levels of trunk AT than Caucasians when adjusted for BMI [17]. Ethnicity was not included as a variable for analysis in the present study.

In the present study males demonstrated larger reduction in motion than females, but also larger initial motion, although the numbers of each sex differed. Sex differences in %AT were also found in this study, similar to those in the literature, with males having higher levels of abdominal VAT [18], and females higher levels of SAT [14]. While sex subgroups were small and of unequal size for analysis in the present study, a sex-based evaluation of %AT and compression effectiveness may be warranted.

Typically, variations of T1W sequences will be used for AT quantification [19], [20], although T2W can also be used [21]. T2W was used in the present study as these are routinely acquired for patients undergoing radiotherapy, however future work will aim to optimise AT quantification sequences using either Dixon or chemical shift imaging.

## Conclusion

This is the first study to assess the impact of BMI and fat measured on MRI on abdominal compression effectiveness. Visceral and subcutaneous fat, and BMI, showed no impact on abdominal compression effectiveness, although reduction of motion was higher in patients with higher BMI. Therefore, abdominal compression should be used on a patient-specific basis and considered alongside other strategies based on motion reduction and dosimetric benefit. Further work is required to identify the impact of patient experience, respiratory kinematics, sex, and overall body composition on motion management, as well as the dosimetric impact of various motion management strategies.

## Informed patient consent

The author(s) confirm that written informed consent has been obtained from the involved patient(s) or if appropriate from the parent, guardian, power of attorney of the involved patient(s); and, they have given approval for this information to be published in this case report (series).

## Funding & acknowledgements

The authors would like to acknowledge Prof. Robert Huddart (Institute of Cancer Research, The Royal Marsden NHS Foundation Trust) for his role as Chief Investigator of the PRIMER study, and Prof. Marcel van Herk (The University of Manchester, The Christie NHS Foundation Trust) for his assistance and provision of access to matching software (MATPEL) during the motion assessment portion of this study.

Mairead Daly is supported by Cancer Research UK RadNet Manchester [C1994/A28701], the Advanced Radiotherapy Technologies Network (ART-NET) [C309/A21993], the NIHR Manchester Biomedical Research Centre (NIHR203308), and The Christie Hospital Charitable Fund. Robert Chuter is supported by Advanced Radiotherapy Technologies Network (ART-NET) [C309/A21993]. Claire Nelder is supported by Cancer Research UK RadNet Manchester [C1994/A28701]. Ananya Choudhury, Alan McWilliam, Lisa McDaid, and Cynthia Eccles are supported by NIHR Manchester Biomedical Research Centre (NIHR203308). The PRIMER study is supported by ICR/RMH NIHR Biomedical Research Centre. This work is also supported by Cancer Research UK Manchester Centre (CTRQQR-2021\100010).

## Declaration of Competing Interest

The authors declare that they have no known competing financial interests or personal relationships that could have appeared to influence the work reported in this paper.
